# Serological Evidence of an Early Seroconversion to Simian Virus 40 in Healthy Children and Adolescents

**DOI:** 10.1371/journal.pone.0061182

**Published:** 2013-04-25

**Authors:** Angelo Taronna, Elisa Mazzoni, Alfredo Corallini, Ilaria Bononi, Silvia Pietrobon, Giovanni Guerra, Caterina Palmonari, Caterina Borgna-Pignatti, Manola Comar, Massimo Bovenzi, Ferruccio Casali, Roberto Marci, Giovanni Rezza, Giuseppe Barbanti-Brodano, Mauro Tognon, Fernanda Martini

**Affiliations:** 1 Section of Microbiology, University of Ferrara, Ferrara, Italy; 2 Section of Cell Biology and Molecular Genetics, University of Ferrara, Ferrara, Italy; 3 Clinical Laboratory Analysis, Ferrara City Hospital, Ferrara, Italy; 4 Clinical Laboratory Analysis, County Hospital Delta, Lagosanto, Italy; 5 Section of Pediatrics, University of Ferrara, Ferrara, Italy; 6 Institute for Maternal and Child Health – Istituto di Ricerca e Cura a Carattere Scientifico “Burlo Garofolo”– Trieste, University of Trieste, Trieste, Italy; 7 Clinical Unit of Occupational Medicine, Department of Medical Sciences, University of Trieste, Trieste, Italy; 8 Clinical Laboratory Analysis, San Marino State Hospital, Borgo Maggiore, Republic of San Marino; 9 Department of Obstetrics and Genecology, University of Ferrara, Ferrara, Italy; 10 Departement of Infectious Diseases, Istituto Superiore di Sanità, Rome, Italy; National Institutes of Health, United States of America

## Abstract

At present Simian virus 40 (SV40) infection in humans appears to be transmitted independently from early contaminated vaccines. In order to test the spread of SV40 infection in children, an immunologic assay employing specific SV40 synthetic peptides corresponding to its viral protein (VP) antigens was employed to estimate the seroprevalence of this polyomavirus in Italian infants and adolescents. Serum samples from 328 children and adolescents, up to 17 years, were investigated. Serum antibodies against SV40 VPs were detected by indirect enzyme-linked immunosorbent assays. The seroprevalence of this polyomavirus was calculated after stratifying the subjects by age. Anti-viral capsid protein 1-2-3 SV40 IgG antibodies were detected in 16% of the study participants. The prevalence of antibodies against SV40 VPs tended to increase with age in children, up to 10 year old (21%). Then, in the cohort of individuals aged 11–17 years, the prevalence decreased (16%). A higher prevalence rate (23%) of SV40 VP antibodies was detected in the cohorts of 1–3 year and 7–10 year old children, than in children and adolescents of the other age groups. This age corresponds to children starting nursery and primary school, respectively, in Italy. IgM antibodies against SV40 VP mimotopes were detected in 6–8 month old children suggesting that SV40 seroconversion can occur early in life. SV40 VP antibodies are present at low prevalence in Italian children (16%), suggesting that SV40 infection, although acquired early in life, probably through different routes, is not widespread. The low SV40 seroprevalence suggests that SV40 is less transmissible than other common polyomaviruses, such as BKV and JCV. Alternatively, our immunologic data could be due to another, as yet undiscovered, human polyomavirus closely related to SV40.

## Introduction

Simian virus 40 (SV40) is a non-enveloped small DNA virus with a genome of approximately 5.2 kb in size. SV40 was recognized in the 1960 as contaminant of both inactivated (Salk) and live (Sabin) anti-poliomyelitis vaccines. After its isolation, SV40 was experimentally characterized as a transforming and oncogenic virus [1], [2]. SV40 late region contains three main genes encoding for three structural polypeptides, the viral capsid proteins 1, 2 and 3 (VP 1-2-3). VP 2 and 3 genes partially overlap [3].

Several studies, carried out mainly by PCR techniques, suggest that SV40 is contagiously transmitted in humans by horizontal infection, independently of the administration of SV40-contaminated vaccines [1], [2]. Moreover, the circulation of SV40 in human populations before the administration of contaminated vaccines cannot be excluded.

SV40 sequences have been detected, at low prevalence and with a low viral DNA load, in blood samples from healthy donors [4], [5], [6] and HIV-negative and HIV-positive patients [4], indicating that human cells are only in part permissive for its multiplication. This observation is in line with the evidence that mesothelial cells [7], [8] immortalized fibroblasts [9] and T-lymphocytes [10] are only semi-permissive SV40 infection in vitro.

SV40 sequences [11], [12], [13], [14], [15] and SV40 antibodies [16], [17] were detected in normal subjects of differing ages, and in patients with different cancer types, including ependymomas, papillary choroid plexus papillomas [18], [19], [20] and bone tumors [21], [22], [23], [24], [25] which are neoplasms at a high incidence in children. It is worth bearing in mind that the association of SV40 with human tumors is not a prove of a causal relation with cancer onset/progression.

A recent WHO/IARC meeting established that, due to a lack of firm evidence, SV40 is not classifiable as a carcinogenic viral agent in humans [26]. The problems concerning the SV40 infection in human populations and its contribution to human cancer was also evaluated by the Immunization Safety Review Committee, established by the Institute of Medicine of the National Academies [27]. The Committee addressed the evidence that epidemiologic studies were flawed by several problems. The Committee recommended the development of specific and sensitive serologic tests to detect SV40 antibodies and the use of standardized techniques which should be accepted and shared by all laboratories involved in SV40 research. Detection SV40 antibodies has been attempted in several studies, using SV40 structural antigens and different serologic methods. However, due to the high protein homology among the three main polyomaviruses, SV40, BK virus (BKV) and JC virus (JCV), the results were always affected by some cross-reactivity [16], [28], [29], [30], [31]. Specific immunologic assays for the identification of SV40-seropositive healthy individuals and serum antibody reactivity to SV40 antigens are of paramount importance in revealing the prevalence of SV40 infection in humans. In particular, little information is available about SV40 infection in children and it is unknown when seroconversion occurs.

In this study, serum samples from healthy children and adolescents were analyzed for exposure to SV40 infection with an immunologic test with synthetic peptides from the SV40 capsid viral protein 1–3 (VPs 1-2-3) epitopes. Since these short synthetic peptides mimic the SV40 VP antigens, they were employed as mimotopes in indirect ELISAs, as recently reported [3], [32] Immunologic data, obtained with these SV40 VP mimotopes, indicate that (i) specific SV40 antibodies can be detected in serum samples from healthy children; (ii) children seroconvert early in life and that (iii) SV40 is circulating in humans, although at a low prevalence and low titer, independently from SV40-contaminated vaccines. It is also possible that our immunologic data could be due to another, as yet undiscovered, human polyomavirus closely related to SV40.

## Results

### SV40 Antibodies Prevalence and Titer in Serum Samples from Healthy Children and Adolescents

In this investigation, additional comparative computer assisted analyses by BLAST program were carried out with the SV40 VP B and C peptides and the corresponding amino acid (aa) sequences of the new human polyomavirus 10 (HPyV10) and hundreds of different BKV and JCV serotypes. Our results indicate a low homology, similar to those already reported for the BKV and JCV prototypes and other polyomaviruses [3]. Data are reported in Text S1 and Tables S1–S6. See the section at the end of the article.

In the first step of this study, indirect ELISA was employed to test serum samples which had been diluted at 1/20 and had been taken from healthy subjects aged from 0.1–17 y old. The samples were tested for reactivity to SV40 epitopes from VP1, VP1 B peptide. Serum samples which reacted with the SV40 VP1 B mimotope reached, in children aged up to one year, the prevalence of 13% for SV40 antibodies in the IgG class, then increased with age, reaching 25% in the 1–10 ys age range. Following this, it remained stable in adolescents, from 11–17 ys of age, with the same prevalence of 25%. The overall prevalence in children and adolescents from 0.1–17 ys of age was 21% (69/328).

**Figure 1:**
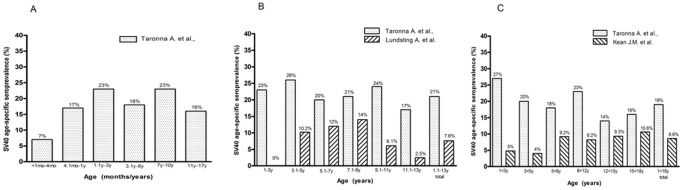
Comparison of SV40 age-specific seroprevalence of antibodies. Comparison of SV40 age-specific seroprevalence of antibodies among 214 Italian children and adolescents, <1–17 year old (this study), Panel A; compared to 288 Swedish children aged between 1 and 13 year old [16] Panel B; and compared to 629 U.S. children and adolescents aged between 1 and 18 year old [17], Panel C.

The same assay was then addressed to detect IgG class serum antibodies against SV40 VP2/3 epitopes, which were present in the VP2/3 C peptide. It turned out that serum samples in the cohort of children, aged 0.1–1 y, reacted with the SV40 VP2/3 C peptide with a prevalence of 11%, which is similar to that detected for the VP1 B peptide. Then, it increased with age with an overall prevalence of 25% detected in the cohort of healthy children aged 1–10 ys, while decreasing to 17% in the cohort of children aged 11–17 ys. Overall, in children aged 0.1–17 ys, the prevalence of antibodies against SV0 peptide VP 2/3 C was 18%. Conversely, seronegative samples for the SV40 VP1 B peptide failed to react with SV40 VP2/3 C epitopes. The exceptions were negligible and were represented by a few serum samples which were found to be negative for VP1 B, while testing positive for VP2/3 C peptide, and vice-versa. The difference was not statistically significant (*P*>0.05) (Table 1; Fig. 1).

**Table 1:** Prevalence of serum IgG antibodies reacting with SV40 VP Mimotopes∧.

Age	Number of Sample	Male %	Number of positive samples (%)		
			VP B	VP C	VPs (B–C)
<1 mo-1 y	114	66	15 (13)	13 (11)	13 (11)*
<1 mo-4 mo	67		5 (7)	5 (7)	5 (7)
4.1 mo-1 y	47		10 (21)	8 (17)	8 (17)
1.1 y-10 ys	126	52	32 (25)	32 (25)	27 (21)
1.1 y-3 ys	35		10 (29)	9 (26)	8 (23)
3.1 ys-6 ys	44		9 (20)	9 (20)	8 (18)
7 ys-10 ys	47		13 (28)	14 (30)	11 (23)
11 ys-17 ys	88	56	22 (25)	15 (17)	14 (16)
1 mo-17 ys	total 328	59	69 (21 )	60 (18)	54 (16)

∧ Human sera were from healthy children and adolescents. Statistical analysis was performed with Chi-square test.

* The different prevalence of SV40 antibodies between the cohort of individuals aged <1 mo-1 year was statistically significant compared to the cohort of subjects aged 1.1–10 years (P = 0.0374).

In this investigation, serum samples were considered SV40-positive when reacting with both VP1 B and VP2/3 C peptides.

The overall prevalence by combining SV40-positive sera, both for VP1 B and VP2/3 C peptides, was 16% (Table 1; Fig. 1). No positive results were obtained with human peptide used as a control, which had an OD of less than 0.1 (0.088–0.098). This OD value is usually consistent with SV40-negative sera.

Serum samples tested by indirect ELISA diluted at 1/20 were considered SV40-positive when above the 0.17–0.19 OD, according to the spectrophotometric reading. Indeed, this cut-off point represents the value that discriminates SV40-negative (sample with OD below 0.17–0.19) from SV40-positive samples (OD above 0.17–0.19). The positive control, represented by the SV40 hyperimmune serum, had an OD of up to 1.8, while the two JCV and BKV hyperimmune sera, which were employed as negative controls, had an OD of less than 0.1. A prevalence selection corresponding to 11%, 21%, and 16%, within the cohort aged from 0.1–17 ys old, was observed in subjects aged 0.1–1 y, 1.1–10 ys, 11–17 ys, respectively. The different prevalence of SV40 antibodies between the cohorts of individuals aged 0.1–1 year (11%) was statistically significant compared with cohort of 1.1–10 years (21%) (P = 0.0374). Interestingly, the prevalence of serum antibodies against SV40 VPs increased with age, with a percentage of 23% in the cohort of children aged 1.1–3 y and 7–10 y old, which is the age in which children start nursery and primary schools, respectively, in Italy (Table 1; Fig. 1). Then, the prevalence declines in the cohort of adolescents 11–17 y old (16%) (Table 1; Fig. 1).

**Figure 2:**
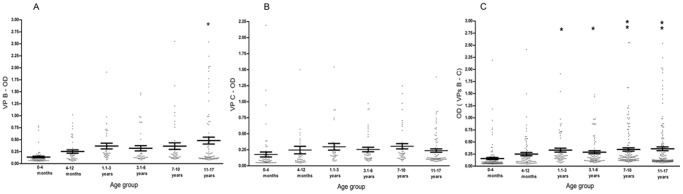
Serologic profile of serum antibody reactivity to SV40 mimotopes, VP1 B (Panel A), VP2/3 C (Panel B) and VPs both peptides B–C (Panel C). Data are presented as values of VPs B and C, OD readings at λ 405 nm, of serum samples diluted at 1∶20 detected in indirect ELISA testing. In scatter dot plotting, each plot represents the dispersion of OD values to a mean level, indicated by the line inside the scatter with standard error of the mean (SEM) for each age group of subjects analyzed. The OD readings of serum samples stratified by age of children were: 0.1–4 months, 4.1–12 months, 1.1–3 ys, 3.1–6 ys, 7–10 ys and 11–17 ys. Data were analyzed with one way Anova analysis, and Newman-Keuls Multiple Comparison Test (OD mean, 95% CI). Panel A. High levels of antibodies against SV40 VP1 B, mean OD values, were observed in children aged 11–17 ys (0.48 OD, 95% CI = 0.34–0.62) vs. children aged 0.1–4 months (0.13 OD, 95% CI = 0.10–0.17) (*P*<0.001) and vs. children aged 4.1–12 months (0.25 OD, CI = 0.18–0.32), with *P*<0.05. Panel B. The different levels of antibodies against SV40VP1 peptide C, mean OD values, were not statistically significant among children. Panel C. High levels of antibodies against SV40VPs, both peptides B and C, mean OD values, were observed in children aged 1.1–3 ys (0.33 OD, CI = 0.25–0.41), and 3.1–6 ys (0.28 OD, CI = 0.22–0.35) vs. children aged 0.1–4 months (0.16 OD, CI = 0.11–0.20), with *P<0.05. Highest level of mean OD values were observed in children aged 7–10 ys (0.35 OD, CI = 0.28–0.41) and 11–17 ys (0.36 OD, CI = 0.28–0.44) vs. children aged 0.1–4 months., with **P<0.001.

Altogether, serum samples tested by indirect ELISA, diluted at 1∶20, had a mean value for both B and C peptides of approximately 0.29 OD, with the highest value of 3.3 OD detected in one serum among those from individuals aged 11–17 y old. Highest level of mean OD values were observed in children aged 7–10 ys (0.35 OD, CI = 0.28–0.41) and 11–17 ys (0.36 OD, CI = 0.28–0.44) vs. children aged <1–4 months (0.15 OD, CI = 0.11–0.19) with **P<0.001. Fig. 2.

The two indirect ELISA tests, with two distinct VP peptides gave overlapping results, thus confirming the presence of antibodies against SV40 VPs in human sera from infants and adolescents (Table 1, Fig. 1). The reduced prevalence of SV40-positive sera in infants aged <1 mo-1 y old could be ascribed to a natural age-dependent immune system that is going to become mature, while the mother’s antibodies are no longer detectable. Indeed, it is well established that the immune system physiologically matures with age, rendering individuals more responsive to infections. An alternative interpretation of the result, as suggested by other studies [16], [17] is that the low prevalence of SV40 antibodies in sera from younger children could depend on the low spread of SV40 infectivity in humans (Fig. 1). Serologic profiles of serum antibody reactivity to SV40 mimotopes are presented in Fig. 2.

### IgM Class Antibodies Against SV40 in Serum Samples

The presence of IgM class antibodies was investigated by indirect ELISA in five IgG SV40-positive sera from children aged 2–8 months. This analysis may indicate when SV40 seroconversion occurs. Two samples from subjects aged 2–4 months were IgM negative, while the other three sera from children aged 6–8 months were IgM positive. This result indicates that SV40 seroconversion can occur in subjects aged 6 months (Table 2).

**Table 2:** Serum antibodies, IgM and IgG class, reacting in indirect ELISA with SV40 VP mimotopes, from five pediatric subjects aged up to 8 months.

Subjects	Age	IgG	IgG*	IgM	IgM*
Code	month	VPs		VPs	
		(OD B;OD C)		(OD B;OD C)	
1D	2	(0.245; 0.316)	+	(0.095;0.096)	−
64B	4	(0.149;0.172)	+	(0.129;0.119)	−
9B	6*	(0.240;0.353)	+	(0.136;0.189)	+
20C	8*	(0.444;0.401)	+	(0.159;0.153)	+
63C	8*	(0.443;0.180)	+	(0.186;0.150)	+

* SV40-positive sera were those samples above the cut-off.

+SV40-positive sample.

−SV40-negative sample.

IgG cut-off value: Peptide B (0.135 OD), Peptide C (0.143 OD).

IgM cut-off value: Peptide B (0.130OD), Peptide C (0.125 OD).

### ELISA with an Automatic Processing System

Forty serum samples from young donors, processed using an automatic system gave the same results as previously obtained by manually performing ELISA. Indeed, indirect ELISA was transferred and repeated on the automatic processing system with the DSX instrument (Dynex Technologies Inc., VI, USA). Specifically, the DSX™ Automated ELISA System is a computer-controlled microplate processing system that fully automates ELISAs. This instrument automates the sample distribution, incubation, reagent addition, washing, and detection phases of microplate assays. This result obtained by this instrument is of interest since it indicates that our serologic tests can be easily transferred to an automatic processing system in common clinical laboratory analyses without result variability.

## Discussion

The prevalence of antibodies to SV40 VPs stratified by age, is shown in Table 1. Overall, 16% of the study population had antibodies against SV40 VPs. The highest relative increase in the proportion of children with antibodies against SV40 VPs was observed in the 1–3 ys and 7–10 ys age group (23%), with more than a 100% increase compared with the age group<1 y old (11%). Subsequently, with age this prevalence declined. Indeed, the prevalence appeared to stabilize for children who were more than 10 y old (16%). It seems that serum antibodies against SV40 have low stability over time. This result is in agreement with previous studies [13], [16]. Indeed, overtime the prevalence of SV40 antibodies does not remain stable in the different cohorts of children and adolescents, as demonstrated by a decreased prevalence in the cohorts of subjects aged 3.1–6 ys and 11–17 ys. The SV40 seroprevalence determined in this study was lower than that recently reported on other 9 human polyomaviruses [17], [33]. The level of SV40 seroprevalence detected in our study suggests that seroconversion can occur at 6 months of age. This finding is consistent with the high proportion of 1- to 3-y old children with high antibody titers, which may be ascribed to a recent infection.

The Italian pattern appears to differ from that determined in studies conducted in Sweden [16] and U.S. [17] that showed a lower prevalence of antibodies against SV40 VP in children. Our results and comparative data are shown in Fig. 1. The difference observed in the results of studies carried out in children from Italy and Sweden, and U.S. may be due to the different ELISA employed. Indeed, in the Swedish and American assays virus like-particles (VLPs) were used as SV40 antigens [16], [17] while in our investigation specific SV40 mimotopes were employed [3], [32]. The SV40 VLPs used in the Swedish and U.S. studies were made up by assemblage of only the VP1 capsid protein [16], [17] while our peptides contain epitopes of both VP1 and VP2/3. The absence of VP2/3 epitopes in VLPs, with consequent lack of recognition of VP2/3 antibodies, may account for the lower percentage of positive sera in those studies [16], [17]. A similar low percentage of adults positive for SV40 antibodies was reported in another study where SV40 VLPs were used as antigens [29]. In this investigation, human sera were adsorbed with BKV and JCV VLPs, thus subtracting a substantial amount of antibodies cross-reacting with SV40 VP epitopes [30]. It has also been proposed that a distinct prevalence of SV40 infection occurs in different countries, that probably relates to different ethnic groups, as well as to distinct hygienic and environmental standards [34], [35].

Our findings suggest that infection appears to be acquired early in life, as a possible consequence of transmission in the family and in community settings. It may be possible that SV40 is transmissible through saliva, urine and stools as occurs for other viruses. For this reason, SV40 transmission may occur when particular environmental conditions prevail, i.e., in the presence of overcrowding and poor hygiene and primarily in areas where the background SV40 prevalence in the general population may be higher, such as in community schools.

In conclusion, our data show that the SV40 infection has spread among Italian children, although at a low prevalence. The presence of SV40 antibodies in children of different ages suggests that distinct routes of transmission may occur [15]. The low prevalence of children exposed to SV40 is in agreement with the low prevalence of SV40-positive individuals among healthy adults, as was shown in previous studies [3], [6], [16], [20], [32], [33], [36]. Immunological data from this study and other investigations suggest that SV40 is also a human virus. Alternatively, it may be that another, as yet undiscovered polyomavirus infect humans. In this case, positive immunologic data could be due to a new virus closely related to SV40.

## Materials and Methods

### Human Samples

Serum samples from children and adolescents (n = 328; male = 194; female = 134) were collected in the 2009–2012 period. Sera were taken from discarded samples in different Institutions in Italy. They were from the Clinical laboratory analysis, Pediatric Section, Obstetrics and Gynecology, University Hospital of Ferrara, Ferrara; University Hospital of Trieste, Trieste; Clinical laboratory analysis, Delta County Hospital, Lagosanto; Clinical laboratory analysis, State Hospital, Republic of San Marino; Dept. Infectious Diseases, Istituto Superiore di Sanità, Rome. Anonymously collected sera were coded with indications of age and gender, only. The project was approved by the County Ethical Committee of Ferrara. Written consent was given by parents of the children and adolescents involved in this study.

### SV40 Mimotopes

Computer assisted analyses allowed us to select 2 specific SV40 peptides, from the late viral region by comparing the three capsid proteins, VP 1-2-3 from SV40, with the amino acids of the human BK (BKV) and JC (JCV) polyomaviruses which are highly homologous to SV40, as well as with other, less homologous polyomaviruses [3]. Previous ELISA results indicated that the two SV40 peptides did not cross-react with the BKV and JCV hyperimmune sera that were employed as controls [3], [32]. The amino acid sequences of the two peptides, known as VP1 B and VP2/3 C, respectively, are as follows:

VP1 B: NH2- NPDEHQKGLSKSLAAEKQFTDDSP- COOH.

VP2/3 C: NH2- IQNDIPRLTSQELERRTQRYLRD- COOH.

VP1 B and VP2/3 C mimotopes were selected as they react specifically in indirect ELISA with the rabbit hyperimmune serum that had been experimentally immunized with SV40 (positive control serum). BKV and JCV hyperimmune sera did not react with VP1 B or VP2/3 C peptides (negative control sera). The amino acid residues of the two specific SV40 VPs, B and C peptides, show low homology with the BKV and JCV VPs [3].

The human peptide hNPS, a.a. sequence SFRNGVGTGMKKTSFQRAKS was employed as a negative control peptide [37]. The synthetic peptides were synthesized using standard procedures and were purchased from UFPeptides s.r.l., Ferrara, Italy [3].

### Control Immune Sera

Hyperimmune sera against SV40 and BKV were obtained in rabbits that had been inoculated with purified viral stocks as previously reported [3]. The serum against JCV was kindly provided by Dr. Major, NIH, Bethesda (MD), U.S.A. [38]. The immune serum anti-BKV was titered using a hemagglutination inhibition (H.A.I.) test employing human erythrocytes group 0, Rh+ [3]. Anti SV40 serum was titered by neutralization assay [3]. Additional SV40-negative human sera, employed as controls, were taken from our previous investigations [3], [32].

### Indirect Enzyme-linked Immunosorbent Assay (ELISA)

Indirect ELISA was developed and standardized to detect specific antibodies against SV40 in human sera using VP1 B VP 2/3 C synthetic peptides [3], [32]. *Peptide coating.* Plates were coated with 5 µg of the selected peptide for each well and diluted in 100 µl of Coating Buffer (Candor Bioscience, Germany) at 4°C for 16 hours. *Peptide blocking.* Blocking was made with 200 µl/well of the Blocking Solution (Candor Bioscience, Germany) at 37°C for 90 min. *Primary antibody adding.* Different wells were covered with 100 µl of serum sample diluted 1/20 Low Cross-Buffer (Candor Bioscience, Germany) containing: positive-control to SV40, represented by immune rabbit serum containing anti-SV40 antibodies, negative controls to BKV and JCV represented by immune sera anti-BKV and anti-JCV, and three human serum samples which were found to be SV40-negative in our previous investigation [3]. Each sample was analyzed three times in duplicate wells. *Secondary antibody adding.* The solution contained a goat anti-human or anti-rabbit IgG heavy and light chain specific peroxidase-conjugate (Calbiochem-Merck, Germany) or peroxidase-labeled affinity purified antibody to human IgM heavy chain μ peroxidase-conjugate (KPL Gaithersburg MD, USA) diluted 1∶10,000 in Low Cross-Buffer [39]. *Dye treatment and spectrophotometric reading.* Samples were treated with 100 µl of 2,2′-azino-bis 3-ethylbenzthiazoline-6-sulfonic acid (ABTS) solution (Sigma-Aldrich, Milan), for 45 min. at RT, and then read on the spectrophotometer (Thermo Electron Corporation, model Multiskan EX, Finland) at a wavelength (λ) of 405 nm. The color intensity in wells where the immunocomplexes were formed was determined by optical density (OD). *Automatic ELISA.* The serologic analysis of human sera, performed by the manual ELISA procedure, was transferred and repeated by an ELISA automatic processing system on the DSX instrument (Dynex Technologies Inc., VI, USA). *Cut-off determination.* The cut-off point was determined in each assay by the value of an OD reading of three negative controls, that was added to the standard deviation multiplied three times (+3SD). Sera with antibodies against SV40 were considered VP-positive upon reacting to both peptides of the late region and when sera that had been analyzed three times by indirect ELISA testing gave the same positive result. SV40 antibody titer was determined by serial dilutions of sera from 1∶20 to 1∶640.

### Cell, Viruses and Neutralization Assay

Viral working stocks were obtained in Vero cells infected with the SV40 776 strain or BKV Gardner strain, as described previously [3]. Permissive CV-1 monkey kidney cells were used for the neutralization assay of SV40 infectivity, as described [3]. BKV and JCV hemagglutination (H.A.) and H.A.I. titrations were carried out as described in detail elsewhere [3].

### Statistical Analysis

Statistical analyses were performed using Prism software (GraphPad, San Diego, CA). Data are presented as a percentage of positive samples. The 95% Confidence Intervals (CI) of the percentage of positive samples are also reported. Differences among proportions were calculated by Chi-square test for independence in the contingency tables.

## Supporting Information

Table S1 **SV40 VP1, peptide B compared to JCV VP1.**
(DOC)

Table S2 **SV40 VP 2/3 peptide C, compared to JCV VP2-3.**
(DOC)

Table S3 **SV40 VP1, peptide B compared to BKV VP1.**
(DOC)

Table S4 **SV40 VP2/3, peptide C compared to BKV VP2-3.**
(DOC)

Table S5 **SV40 VP1 peptide B compared to HPyV10 VP1.**
(DOC)

Table S6 **SV40 VP2/3 peptide C compared to HPyV10 VP2-3.**
(DOC)

Text S1 **Comparative homology analyses of amino acid sequences of SV40 VP1 B and VP2/3 C peptides with the corresponding JCV, BKV and HPyV10 VPs.**
(DOC)

## References

[1] Barbanti-Brodano G, Sabbioni S, Martini F, Negrini M, Corallini A, et al (2004) Simian virus 40 infection in humans and association with human diseases: results and hypotheses. Virology 318: 1–9. 15015494 10.1016/j.virol.2003.09.004

[2] Martini F, Corallini A, Balatti V, Sabbioni S, Pancaldi C, et al (2007) Simian virus 40 in humans. Infect Agent Cancer 2: 13. 17620119 10.1186/1750-9378-2-13 PMC1941725

[3] Corallini A, Mazzoni E, Taronna A, Manfrini M, Carandina G, et al (2012) Specific antibodies reacting with simian virus 40 capsid protein mimotopes in serum samples from healthy blood donors. Hum Immunol 73: 502–510. 22387152 10.1016/j.humimm.2012.02.009

[4] Martini F, Dolcetti R, Gloghini A, Iaccheri L, Carbone A, et al (1998) Simian-virus-40 footprints in human lymphoproliferative disorders of HIV- and HIV+ patients. Int J Cancer 78: 669–674. 9833757 10.1002/(sici)1097-0215(19981209)78:6<669::aid-ijc1>3.0.co;2-b

[5] Dolcetti R, Martini F, Quaia M, Gloghini A, Vignocchi B, et al (2003) Simian virus 40 sequences in human lymphoblastoid B-cell lines. J Virol 77: 1595–1597. 12502874 10.1128/JVI.77.2.1595-1597.2003 PMC140833

[6] Pancaldi C, Balatti V, Guaschino R, Vaniglia F, Corallini A, et al (2009) Simian virus 40 sequences in blood specimens from healthy individuals of Casale Monferrato, an industrial town with a history of asbestos pollution. J Infect 58: 53–60. 19070904 10.1016/j.jinf.2008.10.014

[7] Bocchetta M, Di Resta I, Powers A, Fresco R, Tosolini A, et al (2000) Human mesothelial cells are unusually susceptible to simian virus 40-mediated transformation and asbestos cocarcinogenicity. Proc Natl Acad Sci U S A 97: 10214–10219. 10954737 10.1073/pnas.170207097 PMC27818

[8] Cacciotti P, Libener R, Betta P, Martini F, Porta C, et al (2001) SV40 replication in human mesothelial cells induces HGF/Met receptor activation: a model for viral-related carcinogenesis of human malignant mesothelioma. Proc Natl Acad Sci U S A 98: 12032–12037. 11572935 10.1073/pnas.211026798 PMC59762

[9] Morelli C, Barbisan F, Iaccheri L, Tognon M (2004) SV40-immortalized human fibroblasts as a source of SV40 infectious virions. Mol Med 10: 112–116. 15702218 10.2119/2004-00037.Morelli PMC1431373

[10] Mazzoni E, Rigolin GM, Alaribe FN, Pancaldi C, Maniero S, et al (2012) Simian virus 40 efficiently infects human T lymphocytes and extends their lifespan. Exp Hematol 40: 466–476. 22421183 10.1016/j.exphem.2012.02.008

[11] Vanchiere JA, Nicome RK, Greer JM, Demmler GJ, Butel JS (2005) Frequent detection of polyomaviruses in stool samples from hospitalized children. J Infect Dis 192: 658–664. 16028135 10.1086/432076 PMC4010313

[12] Butel JS, Arrington AS, Wong C, Lednicky JA, Finegold MJ (1999) Molecular evidence of simian virus 40 infections in children. J Infect Dis 180: 884–887. 10438386 10.1086/314915

[13] Butel JS, Jafar S, Wong C, Arrington AS, Opekun AR, et al (1999) Evidence of SV40 infections in hospitalized children. Hum Pathol 30: 1496–1502. 10667429 10.1016/s0046-8177(99)90173-9

[14] Vanchiere JA, White ZS, Butel JS (2005) Detection of BK virus and simian virus 40 in the urine of healthy children. J Med Virol 75: 447–454. 15648074 10.1002/jmv.20287

[15] Patel NC, Vilchez RA, Killen DE, Zanwar P, Sroller V, et al (2008) Detection of polyomavirus SV40 in tonsils from immunocompetent children. J Clin Virol 43: 66–72. 18539521 10.1016/j.jcv.2008.04.011 PMC2584969

[16] Lundstig A, Eliasson L, Lehtinen M, Sasnauskas K, Koskela P, et al (2005) Prevalence and stability of human serum antibodies to simian virus 40 VP1 virus-like particles. J Gen Virol 86: 1703–1708. 15914848 10.1099/vir.0.80783-0

[17] Kean JM, Rao S, Wang M, Garcea RL (2009) Seroepidemiology of human polyomaviruses. PLoS Pathog 5: e1000363. 19325891 10.1371/journal.ppat.1000363 PMC2655709

[18] Bergsagel DJ, Finegold MJ, Butel JS, Kupsky WJ, Garcea RL (1992) DNA sequences similar to those of simian virus 40 in ependymomas and choroid plexus tumors of childhood. N Engl J Med 326: 988–993. 1312224 10.1056/NEJM199204093261504

[19] Lednicky JA, Garcea RL, Bergsagel DJ, Butel JS (1995) Natural simian virus 40 strains are present in human choroid plexus and ependymoma tumors. Virology 212: 710–717. 7571441 10.1006/viro.1995.1529

[20] Martini F, Iaccheri L, Lazzarin L, Carinci P, Corallini A, et al (1996) SV40 early region and large T antigen in human brain tumors, peripheral blood cells, and sperm fluids from healthy individuals. Cancer Res 56: 4820–4825. 8841004

[21] Carbone M, Rizzo P, Procopio A, Giuliano M, Pass HI, et al (1996) SV40-like sequences in human bone tumors. Oncogene 13: 527–535. 8760294

[22] Lednicky JA, Stewart AR, Jenkins JJ 3rd, Finegold MJ, Butel JS (1997) SV40 DNA in human osteosarcomas shows sequence variation among T-antigen genes. Int J Cancer 72: 791–800. 9311596 10.1002/(sici)1097-0215(19970904)72:5<791::aid-ijc15>3.0.co;2-c

[23] Yamamoto H, Nakayama T, Murakami H, Hosaka T, Nakamata T, et al (2000) High incidence of SV40-like sequences detection in tumour and peripheral blood cells of Japanese osteosarcoma patients. Br J Cancer 82: 1677–1681. 10817503 10.1054/bjoc.2000.1213 PMC2374501

[24] Gamberi G, Benassi MS, Pompetti F, Ferrari C, Ragazzini P, et al (2000) Presence and expression of the simian virus-40 genome in human giant cell tumors of bone. Genes Chromosomes Cancer 28: 23–30. 10738299 10.1002/(sici)1098-2264(200005)28:1<23::aid-gcc3>3.0.co;2-w

[25] Martini F, Lazzarin L, Iaccheri L, Vignocchi B, Finocchiaro G, et al (2002) Different simian virus 40 genomic regions and sequences homologous with SV40 large T antigen in DNA of human brain and bone tumors and of leukocytes from blood donors. Cancer 94: 1037–1048. 11920474

[26] Bouvard V, Baan R, Grosse Y, Lauby-Secretan B, El Ghissassi F, et al.. (2012) Carcinogenicity of malaria and of some polyomaviruses. The Lancet Oncology 13 339–340. 10.1016/s1470-2045(12)70125-0 22577663

[27] Stratton K, Almario DA, McCormick MC, editors (2003) Immunization Safety Review:SV40 Contamination of Polio Vaccine and Cancer. Washington, D. C.: The National Academies Press. 25057632

[28] Viscidi RP, Rollison DE, Viscidi E, Clayman B, Rubalcaba E, et al (2003) Serological cross-reactivities between antibodies to simian virus 40, BK virus, and JC virus assessed by virus-like-particle-based enzyme immunoassays. Clin Diagn Lab Immunol 10: 278–285. 12626455 10.1128/CDLI.10.2.278-285.2003 PMC150538

[29] Carter JJ, Madeleine MM, Wipf GC, Garcea RL, Pipkin PA, et al (2003) Lack of serologic evidence for prevalent simian virus 40 infection in humans. J Natl Cancer Inst 95: 1522–1530. 14559874 10.1093/jnci/djg074

[30] Barbanti-Brodano G, Corallini A, Accolla RS, Martini F, Tognon M (2004) Re: Lack of serologic evidence for prevalent simian virus 40 infection in humans. J Natl Cancer Inst 96: 803–804; author reply 804–805. 10.1093/jnci/djh151 15150312

[31] Ribeiro T, Fleury MJ, Granieri E, Castellazzi M, Martini F, et al (2010) Investigation of the prevalence of antibodies against neurotropic polyomaviruses BK, JC and SV40 in sera from patients affected by multiple sclerosis. Neurol Sci 31: 517–521. 20552238 10.1007/s10072-010-0353-y

[32] Mazzoni E, Corallini A, Cristaudo A, Taronna A, Tassi G, et al (2012) High prevalence of serum antibodies reacting wuth simian virus 40 capsid protein minotopes in patients affected by malignant pleural mesothelioma. Proc Natl Acad Sci U S A 109: 18066–18071. 23071320 10.1073/pnas.1213238109 PMC3497789

[33] Nicol JT, Robinot R, Carpentier A, Carandina G, Mazzoni E, et al.. (2013) Age-specific seroprevalence of Merkel cell polyomavirus, Human polyomaviruses 6, 7 and 9 and Trichodysplasia Spinulosa-associated polyomavirus. Clin Vaccine Immunol. 10.1128/CVI.00438-12 PMC3592346 23302741

[34] Butel J (2012) Polyomavirus SV40: Model Infectious Agent of Cancer. In: Robertson ES, editor. Cancer Associated Viruses Current Cancer Research ID1.Perelman School of Medicine, University of Pennsylvania: Springer US. 377–417.

[35] Butel JS (2012) Patterns of polyomavirus SV40 infections and associated cancers in humans: a model. Curr Opin Virol 2: 508–514. 22771310 10.1016/j.coviro.2012.06.004 PMC3422415

[36] Jafar S, Rodriguez-Barradas M, Graham DY, Butel JS (1998) Serological evidence of SV40 infections in HIV-infected and HIV-negative adults. J Med Virol 54: 276–284. 9557293 10.1002/(sici)1096-9071(199804)54:4<276::aid-jmv7>3.0.co;2-1

[37] Guerrini R, Salvadori S, Rizzi A, Regoli D, Calo G (2010) Neurobiology, pharmacology, and medicinal chemistry of neuropeptide S and its receptor. Med Res Rev 30: 751–777. 19824051 10.1002/med.20180

[38] Major EO, Miller AE, Mourrain P, Traub RG, de Widt E, et al (1985) Establishment of a line of human fetal glial cells that supports JC virus multiplication. Proc Natl Acad Sci U S A 82: 1257–1261. 2983332 10.1073/pnas.82.4.1257 PMC397234

[39] Randhawa P, Bohl D, Brennan D, Ruppert K, Ramaswami B, et al (2008) longitudinal analysis of levels of immunoglobulins against BK virus capsid proteins in kidney transplant recipients. Clin Vaccine Immunol 15: 1564–1571. 18753339 10.1128/CVI.00206-08 PMC2565927

