# Supplementary material for: Serological Evidence of an Early Seroconversion to Simian Virus 40 in Healthy Children and Adolescents
Source: PLoS One. 2013 Apr 25;8(4):e61182. doi: 10.1371/journal.pone.0061182 (PMC3636242; doi:10.1371/journal.pone.0061182)
Supplement: Table S2 — SV40 VP 2/3 peptide C, compared to JCV VP2-3. (DOC) [file pone.0061182.s002.doc]

| **Table S2: SV40 VP 2/3 peptide C, compared to JCV VP2-3** | | | | | |
| --- | --- | --- | --- | --- | --- |
|  |  |  |  |  |  |
| **SV40 VP2/3 C** | IQNDIPRLTSQELERRTQRYLRD |  |  |  |  |
| **JCV VP2/3**  **serotype** | **aa sequence** |  | **%**  **homology** | **sequence analyzed** | **Accession Number** |
| A | VRDDLPALTSQEIQRRTQK | * | 58% | 15 | AB183152, AB048564, AB048576, AB048566, AB074582,  AB074583, AB048568, AB048575, AB074588, AB038251,  AB074581, AB048574, AB048567, AB048563, AB074580. |
| B | VRDDLPSLTSQEIQRRTQK | § | 58% | 91 | AB048579, AB118657, AB118659, AB077872, AB198943,  AB048549, AB077873, AB118654, AB048550, AB048560,  AB118655, AB262396, AB048551, AB262397, AB048552,  AB048562, AB048582, AB081028, AB118652, AB118653,  AB048557, AB126991, AB048580, AB198949, AB113138,  AB048548, AB198951, AB077871, AB262398, AB048553,  AB262400, AB127008, AB127010, AB077855, AB077856,  AB113141, AB262405, AB127026, AB127012, AB127014,  AB126984, AB048547, AB126985, AB126986, AB126993,  AB126996, AB127006, AB126998, AB048558, AB198953,  AB048559, AB127001, AB048554, AB126983, AB126997, AB127005, AB127011, AB127013, AB127015, AB127016, AB127017, AB127004, AB127007, AB126981, AB127027, AB126995, AB127018, AB127019, AB126981, AB127009,  AB126994, AB081029, AB077866, AB077858, AB077867,  AB198942, AB262399, AB198954, AB198952, AB077879,  AB198950, AB372036, AF004349, AB118656, AB048561,  AB118651, AB118658, AB048555, AB048556, AB372037, AB126992. |
|  | VRDDLPALTSQEIQRRTQK | * | 58% | 1 | AB074584. |
| C | VRDDLPSLTSQEIQRRTQK | § | 58% | 5 | AB038252, AB038253, AB048545, AB048546, AB198940. |
| TOTAL |  |  |  | 112 |  |
|  |  |  |  |  |  |
| *with the same sequence; § the same sequence; underscored: aa conserved; marked in grey: aa substitution compared to JCV aa sequence, serotype A | | | | | |
