# Supplementary material for: Serological Evidence of an Early Seroconversion to Simian Virus 40 in Healthy Children and Adolescents
Source: PLoS One. 2013 Apr 25;8(4):e61182. doi: 10.1371/journal.pone.0061182 (PMC3636242; doi:10.1371/journal.pone.0061182)
Supplement: Table S3 — SV40 VP1, peptide B compared to BKV VP1. (DOC) [file pone.0061182.s003.doc]

| **Table S3: SV40 VP1, peptide B compared to BKV VP1** | | | | | |
| --- | --- | --- | --- | --- | --- |
|  |  |  |  |  |  |
| **SV40 VP1, B** | NPDEHQKGLSKSLAAEKQFTDDSP |  |  |  |  |
| **BKV**  **serotype** | **aa sequence** |  | **%**  **homology** | **sequences analyzed** | **Accession Number** |
| I | DPDENLRGFSLKLSAENDFSSDSP |  | 50% | 99 | V01109, V01108, DQ989812, DQ98980, DQ989802,  DQ305492, AB263938, AB263928, AB263926, AB263914,  AB263913, AB263912, DQ989806, AY628233, AY628232,  AY628231, AY628230, AY628229, AY628228, AY628227,  AY628226, AY628225, AY628224, AB301095, AB301090,  AB298947, AB263934, AB263932, AB263929, AB263927,  A, DQ989798B263922, AB211374, AB211373, AB211369,  DQ989811, DQ989810, DQ989808, DQ989805, DQ989803,  DQ989801, DQ989800, DQ989799, DQ989797, DQ989795,  DQ989794, AY628236, AY628236, AY628235, AY628234,  AB301100, AB301096, AB301094, AB301093, AB301092,  AB301091, AB301089, AB301087, AB301086, AB263936,  AB263935, AB263925, AB263924, AB263923, AB263921,  AB263919, AB263918, AB263917, AB263915, AB260032,  AB260031, AB260029, AB260028, AB211370, AB301549,  AB301102, A, AB211384B298946, AB298945, AB298942,  AB298941, AB263931, AB263930, AB21792, AB217920,  AB217917, AB211385, AB211383, AB211382, AB21138,  AB211379, AB211378, AB211376, AB211376, AB211375,  AB211372, AB301098, AB301103, AB301088, AB213487. |
|  | DSDENLRGFSLKLSAENDFSSDSP |  | 45 | 1 | DQ989804. |
|  | DPDENLRGFSLKLSAEYDFSSDSP |  | 50% | 1 | DQ989813. |
|  | DPDENLRGFSLKLSAKNDFSSDSP |  | 46% | 3 | DQ989809, AB211371, DQ989796. |
|  | DPDENLRGFSLKLSAQNDFSSDSP |  | 46% | 2 | AY628238, AY628237. |
|  | DPDENLRGFSLKVSAENDFSSDSP |  | 46% | 1 | AB260030. |
|  | DPDKNLRGFSLKLSAENDFSSDSP |  | 46% | 1 | AB217918. |
|  | DPDENLRGFSLKLSVENDFSSDSP |  | 46% | 1 | AB211377. |
| II | DPDDNLRGYSLKLTAENAFDSDSP |  | 46% | 2 | AB30110, EF376992. |
|  | DPDNDLRGYSLKLTAENAFDSDSP |  | 46% | 1 | AB263920. |
|  | - PDENLRGYSLKLTAENAFDSDSP |  | 52% | 1 | AB263916. |
| III | - PDDNLRGYSQHLSAENAFESDSP |  | 48% | 1 | M23122. |
|  | DPDDHLRGYSQHLTAENAFDSDSP |  | 50% | 1 | AB211386. |
| IV | DPDNDLRGYSLRLTAETAFESDSP |  | 46% | 7 | AB269869, AB269860, AB269859, AB269842, AB269832,  AB269831, AB269830. |
|  | DPDNDLRGYSLRLTAETAFDSDSP |  | 46% | 42 | AB269868, AB269862, AB269845, AB269841, AB269826,  AB211389, AB269840, AB269837, AB269836, AB217919, AB211391, AB269851, AB269838, AB269834, AB211388, AB211387, AB269867, AB269865, AB269864, AB269863,  AB269861, AB269858, AB269856, AB269855, AB269854,  AB269853, AB269852, AB269850, AB269846, AB269844,  AB269843, AB269828, AB269827, AB301097, AB269866,  AB269857, AB269849, AB269848, AB269847, AB269829,  AB269825, AB260033, AB269824. |
|  | DPDENLRGFSLRLSAENDFSSDSP |  | 50% | 1 | AB254349. |
|  | DPDNDLRGYSLRLTAETAFNSDSP |  | 46% | 1 | AB211390. |
| TOTAL |  |  |  | 166 |  |
|  |  |  |  |  |  |
| underscored: aa conserved; marked in grey: aa substitution compared to the most frequent BKV sequences | | | | | |
