# Supplementary material for: Serological Evidence of an Early Seroconversion to Simian Virus 40 in Healthy Children and Adolescents
Source: PLoS One. 2013 Apr 25;8(4):e61182. doi: 10.1371/journal.pone.0061182 (PMC3636242; doi:10.1371/journal.pone.0061182)
Supplement: Table S4 — SV40 VP2/3, peptide C compared to BKV VP2-3. (DOC) [file pone.0061182.s004.doc]

| **Table S4: SV40 VP2/3, peptide C compared to BKV VP2-3** | | | | | |
| --- | --- | --- | --- | --- | --- |
|  |  |  |  |  |  |
| **SV40 VP2/3 C** | IQNDIPRLTSQELERRTQRYLRD |  |  |  |  |
| **BKV**  **Serotype** | **aa sequence** |  | **%**  **homology** | **sequences analyzed** | **Accession Number** |
| I | IRDDIPAITSQELQRRTERFFRD | * | 65% | 94 | DQ989813, DQ989809, DQ989806, AY628238, AB263922,  AB211374, AB211373, AB211371, AB211369, AY628224,  AB301099, DQ989811, DQ989810, DQ989808, DQ989805,  DQ989803, DQ989801, DQ989800, DQ989799, DQ989798,  DQ989797, DQ989796, DQ989795, AY628237, DQ989794,  AY628236,AY628235, AY628234, AB301100, AB301096,  AY628232, AY628231, AY628230, AB301094, AB301093,  AB301092, AB301091, AB301089, AB301087, AB301086,  AB263936, AB263935, AB263924, AB263923, AY628229,  AY628228, AY628227, AY628226, AY628225, AB263921,  AB217921, AB263919, AB263918, AB263917, AB263915,  AB260032, AB260031, AB217917, AB211385, AB211384,  AB211383, AB211382, AB211381, AB211379, AB211378,  AB211377, AB211376, AB211375, AB211372, AB301098,  AB301103, AB301088, AB213487, AY628224, AB301095,  AB301090, AB298947, AB263934, AB263932, AB263929,  AB263927, AB263925, AB260030, AB260029, AB260028,  AB211370, AB301102, AB298946, AB298945, AB298942,  AB298941, AB263931, AB263930, AB217918. |
|  | IRDDIPSITSQELQRRTERFFRD |  | 65% | 13 | V01109, V01108, DQ989812, DQ989807, DQ989804,  DQ989802, DQ305492, AB263938, AB263928,  AB263926, AB263914, AB263913, AB263912. |
|  | IRDDIPAMTSQELQRRTERFFRD |  | 65% | 1 | [AY628233](http://www.ncbi.nlm.nih.gov/nucleotide/48869524?report=genbank&log$=nucltop&blast_rank=31&RID=CP66TFXA114) |
|  | IRDDIPAIT - QELQRRTERFFRD |  | 61% | 1 | [AB217920](http://www.ncbi.nlm.nih.gov/nucleotide/83281151?report=genbank&log$=nucltop&blast_rank=52&RID=CP6X4W6E11N) |
| II | IRDDIPAITSQELQRRTERFFRD | * | 65% | 4 | AB301101, AB263920, AB263916, EF376992. |
| III | IRDDIPAITSQELQRRTERFFRD | * | 65% | 2 | M23122, AB211386. |
| IV | IRDDIPAITSQELQRRTERFFRD | * | 65% | 50 | AB269869, AB269860, AB269859, AB269842, AB269868,  AB269862, AB269845, AB269841, AB269826, AB211389, AB269840, AB269837, AB269836, AB217919, AB211391,  AB211390, AB269851, AB269838, AB269834, AB211388,  AB211387, AB269867, AB269865, AB269864, AB269863,  AB269861, AB269858, AB269856, AB269855, AB269854,  AB269853, AB269852, AB269846, AB269844, AB269843,  AB269828, AB269827, AB301097, AB269866, AB269857,  AB269849, AB269848, AB269847, AB269832, AB269831,  AB269830, AB269829, AB269825, AB260033, AB269824. |
|  | IRDDIPAITSHELQRRTERFFRD |  | 61% | 1 | [AB269850](http://www.ncbi.nlm.nih.gov/nucleotide/154800236?report=genbank&log$=nucltop&blast_rank=80&RID=CP66TFXA114) |
| TOTAL |  |  |  | 166 |  |
|  |  |  |  |  |  |
| *with the same sequence; underscored: aa conserved; marked in grey: aa substitution compared to the most frequent BKV sequences | | | | | |
