# Supplementary material for: Serological Evidence of an Early Seroconversion to Simian Virus 40 in Healthy Children and Adolescents
Source: PLoS One. 2013 Apr 25;8(4):e61182. doi: 10.1371/journal.pone.0061182 (PMC3636242; doi:10.1371/journal.pone.0061182)
Supplement: Table S5 — SV40 VP1 peptide B compared to HPyV10 VP1. (DOC) [file pone.0061182.s005.doc]

| **Table S5: SV40 VP1 peptide B compared to HPyV10 VP1** | | | | | |
| --- | --- | --- | --- | --- | --- |
|  |  |  |  |  |  |
| **SV40 VP1 B** | NPDEHQKGLSKSLAAEKQFTDDSP |  |  |  |  |
| **HpyV10 serotype** | **aa sequence** |  | **% homology** | **sequence**  **analyzed** | **Accession Number** |
| HpyV10 | NDVKTNKWYGYS - - - DPITVTNTP |  | 16% | 4 | JX262162, JX259273, JQ898291, JQ898292 |
| underscored: aa conserved | |  |  |  |  |
