# Supplementary material for: Serological Evidence of an Early Seroconversion to Simian Virus 40 in Healthy Children and Adolescents
Source: PLoS One. 2013 Apr 25;8(4):e61182. doi: 10.1371/journal.pone.0061182 (PMC3636242; doi:10.1371/journal.pone.0061182)
Supplement: Table S6 — SV40 VP2/3 peptide C compared to HPyV10 VP2-3. (DOC) [file pone.0061182.s006.doc]

| **Table S6: SV40 VP2/3 peptide C compared to HPyV10 VP2-3** | | | | | | |
| --- | --- | --- | --- | --- | --- | --- |
|  |  |  |  |  | |  |
| **SV40 VP2/3 C** | IQNDIPRLTSQELERRTQRYLRD |  |  |  | |  |
| **HpyV10 serotype** | **aa sequence** |  | **%**  **homology** | **sequence analyzed** | **Accession Number** | |
| HpyV10 | - -NTARLLASSAVDNVYNVGEQG |  | 13% | 3 | | JX262162, JX259273, JQ898292 |
|  | LLSET-RQTARLLASNAVDNVY NVGEQGLQN -IQN |  | 22% | 1 | | JQ898291 |
| TOTAL |  |  |  | 4 | |  |

underscored: aa conserved; marked in grey: aa substitution compared to the most frequent HpyV10 sequences
