# Supplementary material for: Serological Evidence of an Early Seroconversion to Simian Virus 40 in Healthy Children and Adolescents
Source: PLoS One. 2013 Apr 25;8(4):e61182. doi: 10.1371/journal.pone.0061182 (PMC3636242; doi:10.1371/journal.pone.0061182)
Supplement: Text S1 — Comparative homology analyses of amino acid sequences of SV40 VP1 B and VP2/3 C peptides with the corresponding JCV, BKV and HPyV10 VPs. (DOC) [file pone.0061182.s007.doc]

**Supporting information.**

**Comparative homology analyses of amino acid sequences of SV40 VP1 B and VP2/3 C peptides with the corresponding JCV, BKV and HPyV10 VPs**

Amino acid (aa) sequence homology (%) of SV40 VP B and VP C peptides was comparatively analyzed, through BLAST program, with 112 JCV , 166 BKV and 4 HPyV10 sequences .

**References**

1. Sugimoto, C., et al., *Typing of urinary JC virus DNA offers a novel means of tracing human migrations.* Proceedings of the National Academy of Sciences of the United States of America, 1997. **94**(17): p. 9191-6.

2. Yogo, Y., et al., *JC virus genotyping offers a new paradigm in the study of human populations.* Reviews in medical virology, 2004. **14**(3): p. 179-91.

3. Pastrana, D.V., et al., *Neutralization serotyping of BK polyomavirus infection in kidney transplant recipients.* PLoS pathogens, 2012. **8**(4): p. e1002650.

4. Luo, C., et al., *Genotyping schemes for polyomavirus BK, using gene-specific phylogenetic trees and single nucleotide polymorphism analysis.* Journal of virology, 2009. **83**(5): p. 2285-97.

5. Yu, G., et al., *Discovery of a novel polyomavirus in acute diarrheal samples from children.* PloS one, 2012. **7**(11): p. e49449.

6. Buck, C.B., et al., *Complete genome sequence of a tenth human polyomavirus.* Journal of virology, 2012. **86**(19): p. 10887.
